# A nationwide survey on participation in cardiac rehabilitation among patients with coronary heart disease using health claims data in Japan

**DOI:** 10.1038/s41598-021-99516-1

**Published:** 2021-10-11

**Authors:** Shosuke Ohtera, Genta Kato, Hiroaki Ueshima, Yukiko Mori, Yuka Nakatani, Neiko Ozasa, Takeo Nakayama, Tomohiro Kuroda

**Affiliations:** 1 Division of Medical Information Technology and Administration Planning, Kyoto University Hospital, 54 Shogoin-kawahara-cho, Sakyo-ku, Kyoto, 606-8507 Japan; 2 Center for Outcomes Research and Economic Evaluation for Health, National Institute of Public Health, 2-3-6 Minami, Wako-shi, Saitama, 351-0197 Japan; 3 Solutions Center for Health Insurance Claims, Kyoto University Hospital, 54 Shogoin-kawahara-cho, Sakyo-ku, Kyoto, 606-8507 Japan; 4 Department of Health Informatics, Kyoto University School of Public Health, Yoshida-Konoe-cho, Sakyo-ku, Kyoto, 606-8501 Japan; 5 Department of Cardiovascular Medicine, Graduate School of Medicine, Kyoto University, 54 Shogoin-kawahara-cho, Sakyo-ku, Kyoto, 606-8507 Japan

**Keywords:** Rehabilitation, Cardiology

## Abstract

Poor implementation and variable quality of cardiac rehabilitation (CR) for coronary heart disease (CHD) have been a global concern. This nationwide study aimed to clarify the implementation of and participation in CR among CHD patients and associated factors in Japan. We conducted a retrospective cohort study using data extracted from the National Database of Health Insurance Claims and Specific Health Checkups of Japan. Patients who underwent percutaneous coronary intervention (PCI) or coronary artery bypass grafting (CABG) in 2017–2018 were included. Aspects of CR were assessed in terms of (1) participation in exercise-based CR, (2) pharmacological education, and (3) nutritional education. Of 87,829 eligible patients, 32% had participated in exercise-based CR, with a mean program length of 40 ± 71 days. CABG was associated with higher CR participation compared to PCI (OR 10.2, 95% CI 9.6–10.8). Patients living in the Kyushu region were more likely to participate in CR (OR 2.59, 95% CI 2.39–2.81). Among patients who participated in CR, 92% received pharmacological education, whereas only 67% received nutritional education. In Japan, the implementation of CR for CHD is insufficient and involved varying personal, therapeutic, and geographical factors. CR implementation needs to be promoted in the future.

## Introduction

Coronary heart disease (CHD) is the top cause of death globally, accounting for approximately 10 million deaths in 2016^1^. In Japan, heart disease is the second leading cause of death; 20% and 24% of deaths are attributable to ischemic heart disease and heart failure, repsectively^2^. Although the widespread use of percutaneous coronary intervention (PCI) has improved short-term outcomes in acute coronary syndrome, including acute myocardial infarction and unstable angina^3,4^, subsequent development of heart failure has been problematic^5–7^. Exercise-based cardiac rehabilitation (CR) for patients with CHD reduces risks of cardiac mortality and hospital admission, and there is mounting evidence that exercise-based CR and patient education are beneficial for improving health-related quality of life^8–10^. Thus, clinical practice guidelines internationally recommend comprehensive CR that includes exercise intervention, control of coronary risk factors, physical activity, and patient education by multidisciplinary professionals, as well as quality measurements, for secondary prevention of cardiovascular diseases^7,11–15^. However, previous studies demonstrated the lack of availability and delivery of CR for eligible patients, suggesting the need for interventions that promote patient utilization of CR, such as face-to-face or technology-based communication by nurses and allied health professionals^16–23^.

In Japan, some studies suggested that CR is underutilized, and that ST-segment-elevation myocardial infarction and coronary artery bypass grafting (CABG) are factors that facilitate patient participation in CR^24–26^. However, no study has clarified the rate of CR participation among CHD patients in Japan at the national level. In this study, we used a national database of health claims data to analyze rates of CR implementation and participation among Japanese patients with CHD, and to explore factors associated with the use of CR based on individual and hospital information.

## Methods

### Study design and data source

We conducted a retrospective cohort study using data extracted from the National Database of Health Insurance Claims and Specific Health Checkups of Japan (NDB), which collects electrical claims records on insured health care services provided to citizens under universal health coverage in Japan, except the portion of public services not covered by universal health care, such as medical assistance for welfare recipients (equivalent to 7% of national health care costs). The database was created for the development, implementation, and evaluation of medical care expenditure regulation plans based on the “Act on Assurance of Medical Care for Elderly People” enacted in April 2008. Researchers and policy makers can acquire NDB data for the purpose of promoting evidence-based policy making with the aim to improve health service quality, in accordance with “the schedule of new information network technique strategy” decided by the Japanese Cabinet in June 2010. The Ministry of Health, Labour and Welfare began providing NDB data in 2011 and original studies using the data have been published in a variety of medical research areas^27–31^. In this study, we accessed data from the NDB On-site Research Center at Kyoto University Hospital. For data cleaning, a patient-matching technique was used according to a previous study^32^. This study was approved by the Ethics Committee of Kyoto University Graduate School and Faculty of Medicine and performed in accordance with the Ethical Guidelines for Medical and Health Research Involving Human Subjects established by the Ministry of Health, Labour and Welfare. The Ethics Committee of Kyoto University Graduate School and Faculty of Medicine waived the requirement for informed consent, since this study used anonymized data provided in accordance with legal provisions.

### Study setting and population

Patients aged ≥ 40 years who underwent PCI or CABG for the first time in the past three years between April 2017 and March 2018 in Japan were included in this study. We determined the onset of CHD in these patients, and followed them for up to one year after PCI/CABG to observe aspects of CR services. The sample size was equivalent to the source population with the above inclusion criteria. We identified health claims indicating the provision of PCI or CABG using billing codes for surgical procedures assigned by the Ministry of Health, Labour and Welfare according to a previous study in Japan^25^. We did not exclude patients who underwent combined coronary bypass grafting and heart valve surgery (replacement or repair). We identified PCI by treatment codes “K546 (percutaneous coronary angioplasty)”, “K547 (percutaneous coronary atherectomy)”, “K548 (percutaneous coronary angioplasty (with special catheter))”, “K549 (percutaneous coronary stenting)”, “K550 (intracoronary thrombolysis)”, “K550-2 (percutaneous coronary artery thrombectomy),” or “K551 (coronary angioplasty (thromboendarterectomy)),” and CABG by “K552 (coronary artery and aortic bypass grafting)” or “K552-2 (coronary artery and aortic bypass grafting (not using artificial heart–lung).”

### Measurements

We assessed patient participation in the three aspects of CR by referring to a previous study that proposed a set of quality indicators for CR in Japan^33^. The first aspect is exercise-based CR within a year after hospitalization for PCI or CABG. We also assessed the duration of participation in CR regardless of whether it was carried out in an inpatient or outpatient setting. In Japan, inpatient CR is prescribed by physicians in acute care hospitals. After discharge, CR services can be continued on an outpatient basis. Home-based CR is currently not reimbursed by national health insurance. Whether patients participate in inpatient CR or outpatient CR, there is no standardized frequency or duration for programs. For the purposes of this study, participation in CR was defined as having attended a program one or more times. Duration of CR (number of dates) was handled as a continuous variable. In addition, the percentage of hospitals that provided CR among those that performed PCI or CABG was calculated by region. The second aspect is pharmacological education, which was determined by reimbursement records from when a pharmacist provided medication instruction or support to inpatients with physician consent, or when a health care professional provided guidance on medication to patients after discharge. The third aspect is nutritional education, which was determined by reimbursement records from when a dietitian provided dietary guidance to inpatients or outpatients under physician instruction. We used medical practice billing codes to identify these aspects, e.g., “180,027,410 (Cardiovascular Disease Rehabilitation Fee (1)” and “180,027,510 (Cardiovascular Disease Rehabilitation Fee (2)” for exercise-based CR.

As covariates, we selected sex, age, type of procedure (PCI or CABG), comorbidities in the past three years before the procedure (diabetes, hypertension, and dyslipidemia, which are coronary risk factors), and the Charlson comorbidity index (CCI)^34^. Participants were divided into 5-year age groups due to the nature of the database. In addition, regarding hospitals where eligible patients underwent PCI or CABG, hospital volume (i.e., the number of patients who undergo PCI or CABG per year) and hospital location (divided into eight regions in Japan) were examined.

### Statistical analysis

Univariate analysis of variables was performed for the three aspects of CR (participation in exercise-based CR, pharmacological education, and nutritional education). Multivariable logistic regression was used to estimate factors associated with the three aspects of CR. R version 3.4.1 was used for all analyses, with p < 0.05 considered significant.

## Results

### Participation in Exercise-based CR

A total of 87,829 patients from 1411 hospitals were eligible for this study, comprising 80,794 (92%) PCI patients and 7035 (8%) CABG patients (Table 1). Mean age was 66.6 ± 10.2 years. In total, 28,159 (32%) patients participated in exercise-based CR within a year after hospital admission due to CHD. Mean program length was 39.9 ± 71.4 days. Among patients who underwent PCI and CABG, 22,554 (28%) and 5605 (80%) participated in CR programs, respectively (Table 2). The rate of participation varied among eight regions where the hospitals were located, ranging from 27.8% (Shikoku) to 45.1% (Kyushu). In a logistic regression model, CABG was associated with higher CR participation compared to PCI (OR 10.2, 95% CI 9.6–10.8), younger age was associated with lower CR participation compared to age ≥ 75 years (OR 0.81, 95%CI 0.78–0.85 for age 65–74 years, OR 0.79, 95% CI 0.76–0.82 for age 40–64 years), and participants in Kyushu and Chugoku regions (western Japan) were more likely to participate in CR (OR 2.59, 95% CI 2.39–2.81 and OR 1.81, 95% CI 1.66–1.98, respectively). The proportion of hospitals providing CR among the target facilities was high in Chugoku and Shikoku regions (Supplementary Table S1).

**Table 1:** Characteristics of eligible patients.

	Eligible patients, N (%)
	N = 87,829
**Age**	
40–64	31,796 (36.2)
65–74	32,453 (37.0)
≥ 75	23,580 (26.8)
**Sex**	
Male	69,929 (79.6)
Female	17,900 (20.4)
**PCI/CABG**	
PCI	80,794 (92)
CABG	7035 (8.0)
**Coronary risk factors**	
**Diabetes**	
Yes	67,753 (77.1)
No	20,076 (22.9)
**Hypertension**	
Yes	30,987 (35.3)
No	56,842 (64.7)
**Dyslipidemia**	
Yes	28,900 (32.9)
No	58,929 (67.1)
**Charlson comorbidity index**	
≤ 2	65,115 (74.1)
3	11,201 (12.8)
≤ 4	11,513 (13.1)
**Hospital information**	
**Patient volume per year**	
< 50	12,832 (14.6)
50–100	20,776 (23.7)
100–150	17,084 (19.5)
150–200	11,757 (13.4)
≥ 200	25,380 (28.9)
**Region**	
Hokkaido	5313 (6.0)
Tohoku	4232 (4.8)
Kanto	32,958 (37.5)
Chubu	13,563 (15.4)
Kinki	17,184 (19.6)
Chugoku	5011 (5.7)
Shikoku	1701 (1.9)
Kyushu	7867 (9.0)

*PCI* percutaneous coronary intervention, *CABG* coronary artery bypass grafting.

**Table 2:** Assessment of participation in exercise-based cardiac rehabilitation (CR).

	Patients who participated in exercise-based CR, n (%)	Patients who did not participate in exercise-based CR, n (%)	Rate of participation in exercise-based CR, %	Logistic regression		Follow-up of exercise-based CR Mean (SD), days
	n = 28,159	n = 59,670		OR (95%CI)a	*P* value	
**Age**						
40–64	10,846 (38.5)	20,950 (35.1)	34.1	0.79 (0.76–0.82)	< 0.01	30.3 (60.7)
65–74	10,129 (36.0)	22,324 (37.4)	31.2	0.81 (0.78–0.85)	< 0.01	41.9 (73.5)
≥ 75	7184 (25.5)	16,396 (27.5)	30.5	Ref		51.9 (80.8)
**Sex**						
Male	22,522 (80.0)	47,407 (79.4)	32.2	Ref		38.1 (69.5)
Female	5637 (20.0)	12,263 (20.6)	31.5	1.00 (0.96–1.04)	0.86	47.4 (78.4)
**PCI/CABG**						
PCI	22,554 (80.1)	58,240 (97.6)	27.9	Ref		40.4 (75.9)
CABG	5605 (19.9)	1430 (2.4)	79.7	10.2 (9.56–10.8)	< 0.01	38.0 (49.8)
**Coronary risk factors**						
**Diabetes**						
Yes	21,405 (76.0)	46,348 (77.7)	31.6	0.94 (0.90–0.97)	< 0.01	40.3 (72.2)
No	6754 (24.0)	13,322 (22.3)	33.6	Ref		38.9 (69.0)
**Hypertension**						
Yes	8834 (31.4)	22,153 (37.1)	28.5	1.27 (1.22–1.31)	< 0.01	41.9 (68.2)
No	19,325 (68.6)	37,517 (62.9)	34	Ref		39.0 (72.9)
**Dyslipidemia**						
Yes	8977 (31.9)	19,923 (33.4)	31.1	1.05 (1.01–1.09)	0.01	46.4 (70.6)
No	19,182 (68.1)	39,747 (66.6)	32.6	Ref		36.9 (71.6)
**Charlson comorbidity index**						
≤ 2	19,410 (68.9)	45,705 (76.6)	29.8	Ref		36.4 (67.7)
3	4523 (16.1)	6678 (11.2)	40.4	1.55 (1.48–1.62)	< 0.01	41.9 (73.7)
≥ 4	4226 (15.0)	7287 (12.2)	36.7	1.22 (1.16–1.28)	< 0.01	54.2 (82.9)
**Hospital information**						
**Patient volume per year**						
< 50	2993 (10.6)	9839 (16.5)	23.3	Ref		44.7 (77.9)
50–100	6699 (23.8)	14,077 (23.6)	32.2	1.50 (1.43–1.58)	< 0.01	40.8 (72.8)
100–150	5826 (20.7)	11,258 (18.9)	34.1	1.64 (1.55–1.73)	< 0.01	36.3 (65.3)
150–200	4112 (14.6)	7645 (12.8)	35.0	1.79 (1.69–1.90)	< 0.01	42.2 (74.9)
≥ 200	8529 (30.3)	16,851 (28.2)	33.6	1.55 (1.48–1.64)	< 0.01	39.0 (70.2)
**Region**						
Hokkaido	1504 (5.3)	3809 (6.4)	28.3	Ref		46.2 (78.2)
Tohoku	1237 (4.4)	2995 (5.0)	29.2	1.18 (1.07–1.30)	< 0.01	31.3 (55.0)
Kanto	9532 (33.9)	23,426 (39.3)	28.9	1.09 (1.02–1.17)	0.02	40.3 (71.4)
Chubu	4459 (15.8)	9104 (15.3)	32.9	1.34 (1.24–1.44)	< 0.01	37.6 (69.5)
Kinki	5483 (19.5)	11,701 (19.6)	31.9	1.34 (1.24–1.44)	< 0.01	42.3 (74.0)
Chugoku	1925 (6.8)	3086 (5.2)	38.4	1.81 (1.66–1.98)	< 0.01	45.0 (76.4)
Shikoku	473 (1.7)	1228 (2.1)	27.8	1.26 (1.11–1.43)	< 0.01	28.3 (56.6)
Kyushu	3546 (12.6)	4321 (7.2)	45.1	2.59 (2.39–2.81)	< 0.01	37.6 (70.4)

*OR* odds ratio, *CI* confidence interval, *SD* standard deviation, *PCI* percutaneous coronary intervention, *CABG* coronary artery bypass grafting.

### Nutritional and pharmacological education

Among CR participants, 25,935 (92.1%) were provided with pharmacological education. CABG (OR:1.26, 95% CI 1.12–1.42), diabetes (OR 1.29, 95% CI 1.15–1.44), and dyslipidemia (OR 1.28, 95% CI 1.16–1.41) were factors associated with higher provision of pharmacological education (Table 3). Nutritional education was provided to 18,743 (66.6%) CR participants. CABG was associated with lower provision of nutritional education (OR 0.66, 95% CI 0.62–0.71) (Table 4).

**Table 3:** Assessment of participation in pharmacological education.

	Patients who participated in pharmacological education, n (%)	Patients who did not participate in pharmacological education, n (%)	Rate of participation in pharmacological education, %	Logistic regression	
	n = 25,935	n = 2224		OR (95%CI)	*P* value
**Age**					
40–64	10,001 (38.6)	845 (38.0)	92.2	0.94 (0.85–1.05)	0.26
65–74	9318 (35.9)	811 (36.5)	92.0	0.90 (0.80–1.02)	0.10
≥ 75	6616 (25.5)	568 (25.5)	92.1	Ref	
**Sex**					
Male	20,744 (80.0)	1778 (79.9)	92.1	Ref	
Female	5191 (20.0)	446 (20.1)	92.1	1.00 (0.89–1.12)	0.98
**PCI/CABG**					
PCI	20,714 (79.9)	1840 (82.7)	91.8	Ref	
CABG	5221 (20.1)	384 (17.3)	93.1	1.26 (1.12–1.42)	< 0.01
**Coronary risk factors**					
**Diabetes**					
Yes	19,642 (75.7)	1763 (79.3)	91.8	1.29 (1.15–1.44)	< 0.01
No	6293 (24.3)	461 (20.7)	93.2	Ref	
**Hypertension**					
Yes	8048 (31.0)	786 (35.3)	91.1	1.11 (1.00–1.23)	0.04
No	17,887 (69.0)	1438 (64.7)	92.6	Ref	
**Dyslipidemia**					
Yes	8133 (31.4)	844 (37.9)	90.6	1.28 (1.16–1.41)	< 0.01
No	17,802 (68.6)	1380 (62.1)	92.8	Ref	
**Charlson comorbidity index**					
≤ 2	17,899 (69)	1511 (67.9)	92.2	Ref	
3	4184 (16.1)	339 (15.2)	92.5	0.95 (0.83–1.08)	0.39
≥ 4	3852 (14.9)	374 (16.8)	91.2	0.76 (0.67–0.86)	< 0.01
**Hospital information**					
**Patient volume per year**					
< 50	2655 (10.2)	338 (15.2)	88.7	Ref	
50–100	6254 (24.1)	445 (20.0)	93.4	1.81 (1.55–2.11)	< 0.01
100–150	5277 (20.3)	549 (24.7)	90.6	1.10 (0.95–1.28)	0.21
150–200	3840 (14.8)	272 (12.2)	93.4	1.54 (1.29–1.84)	< 0.01
≥ 200	7909 (30.5)	620 (27.9)	92.7	1.48 (1.27–1.71)	< 0.01
**Region**					
Hokkaido	1375 (5.3)	129 (5.8)	91.4	Ref	
Tohoku	986 (3.8)	251 (11.3)	79.7	0.35 (0.27–0.44)	< 0.01
Kanto	8707 (33.6)	825 (37.1)	91.3	1.02 (0.84–1.24)	0.82
Chubu	4172 (16.1)	287 (12.9)	93.6	1.44 (1.15–1.79)	< 0.01
Kinki	5210 (20.1)	273 (12.3)	95.0	1.83 (1.46–2.27)	< 0.01
Chugoku	1849 (7.1)	76 (3.4)	96.1	2.37 (1.77–3.19)	< 0.01
Shikoku	441 (1.7)	32 (1.4)	93.2	1.46 (0.98–2.22)	0.07
Kyushu	3195 (12.3)	351 (15.8)	90.1	0.88 (0.70–1.08)	0.23

*OR* odds ratio, *CI* confidence interval, *PCI* percutaneous coronary intervention, *CABG* coronary artery bypass grafting.

**Table 4:** Assessment of participation in nutritional education.

	Patients who participated in nutritional education, n (%)	Patients who did not participate in nutritional education, n (%)	Rate of participation in nutritional education, %	Logistic regression	*P* value
	n = 18,743	n = 9416		OR (95%CI)	
**Age**					
40–64	7868 (42.0)	2978 (31.6)	72.5	0.85 (0.80–0.90)	< 0.01
65–74	6866 (26.5)	3263 (34.7)	67.8	0.60 (0.56–0.70)	< 0.01
≥ 75	4009 (15.5)	3175 (33.7)	55.8	Ref	
**Sex**					
Male	15,232 (58.7)	7290 (77.4)	67.6	0.92 (0.86–1.00)	0.01
Female	3511 (13.5)	2126 (22.6)	62.3	Ref	
**PCI/CABG**					
PCI	15,557 (60.0)	6997 (74.3)	69.0	Ref	
CABG	3186 (12.3)	2419 (25.7)	56.8	0.66 (0.62–0.70)	< 0.01
**Coronary risk factors**					
**Diabetes**					
Yes	14,146 (54.5)	7259 (77.1)	66.1	1.17 (1.10–1.20)	< 0.01
No	4597 (17.7)	2157 (22.9)	68.1	Ref	
**Hypertension**					
Yes	5491 (21.2)	3343 (35.5)	62.2	1.22 (1.15–1.30)	< 0.01
No	13,252 (51.1)	6073 (64.5)	68.6	Ref	
**Dyslipidemia**					
Yes	5296 (20.4)	3681 (39.1)	59.0	1.40 (1.32–1.50)	< 0.01
No	13,447 (51.8)	5735 (60.9)	70.1	Ref	
**Charlson comorbidity index**					
≤ 2	13,253 (51.1)	6157 (65.4)	68.3		
3	3009 (11.6)	1514 (16.1)	66.5	0.88 (0.82–1.00)	< 0.01
≥ 4	2481 (9.6)	1745 (18.5)	58.7	0.69 (0.64–0.70)	< 0.01
**Hospital information**					
**Patient volume per year**					
< 50	2149 (8.3)	844 (9.0)	71.8	Ref	
50–100	4634 (17.9)	2065 (21.9)	69.2	0.95 (0.87–1.10)	0.36
100–150	3787 (14.6)	2039 (21.7)	65.0	0.89 (0.81–1.00)	0.03
150–200	2863 (11.0)	1249 (13.3)	69.6	1.13 (1.01–1.30)	0.03
≥ 200	5310 (20.5)	3219 (34.2)	62.3	0.87 (0.79–1.00)	< 0.01
**Region**					
Hokkaido	1007 (3.9)	497 (5.3)	67.0	Ref	
Tohoku	911 (3.5)	326 (3.5)	73.6	1.25 (1.05–1.50)	0.01
Kanto	5561 (21.4)	3971 (42.2)	58.3	0.76 (0.67–0.90)	< 0.01
Chubu	3252 (12.5)	1207 (12.8)	72.9	1.31 (1.15–1.50)	< 0.01
Kinki	3821 (14.7)	1662 (17.7)	69.7	1.18 (1.04–1.30)	0.01
Chugoku	1378 (5.3)	547 (5.8)	71.6	1.30 (1.12–1.50)	< 0.01
Shikoku	380 (1.5)	93 (1.0)	80.3	1.69 (1.31–2.20)	< 0.01
Kyushu	2433 (9.4)	1113 (11.8)	68.6	0.96 (0.84–1.10)	0.59

*OR* odds ratio, *CI* confidence interval, *PCI* percutaneous coronary intervention, *CABG* coronary artery bypass grafting.

## Discussion

This study clarified the implementation of and participation in CR among patients who underwent PCI or CABG between April 2017 and March 2018 in Japan using nationwide claims data. Only 32% of eligible patients participated in exercise-based CR, with a mean program length of 40 days, despite that both inpatients and outpatients were included in the analysis. CABG was associated with a higher odds of CR participation compared to PCI, and more hospitals in the western part of Japan (i.e., Kyushu and Chugoku regions) performed CR. Moreover, among CR participants, 92% were provided with pharmacological education, whereas only 67% were provided with nutritional education.

Previous studies examined the status of CR referral and suggested that factors such as acute myocardial infarction, cardiac surgery, accessibility to hospitals, hospitals with greater PCI volume, and hospitals that perform cardiovascular procedures were associated with increased referrals^18,20,24–26^. In addition, waiting time from referral to enrollment and follow-up sessions were measured as aspects of participation in CR after referral in some studes^35–37^. However, the nationwide status of CR participation has not been clarified in Japan.

The present study revealed a higher rate of CR participation among patients who underwent PCI or CABG than that reported in a previous study (24%) focused on a younger Japanese population aged ≤ 65 years^25^. We consider this to be reasonable, given that our multivariable regression model estimated younger age to be associated with a lower odds of CR participation. Nevertheless, as has been pointed out previously, this level of participation is lower compared to other countries^38^. In addition, while previous studies in North America reported that men are more likely to participate in CR than women, the present study found no difference between men and women. This finding, however, is consistent with the results of previous studies in Japan^25,39–41^. We also found that patients who underwent CABG were more likely to participate in CR than those who underwent PCI, consistent with a previous report^25^. Some regions in the western part of Japan performed CR more than other regions, showing geographical variation in the implementation of CR as previously reported in the United States^18,20^. Our results also suggested that the supply of CR programs is higher in the western part of Japan, which is in line with previous reports that the supply of medical care in Japan might be in a state of "west high, east low"^42,43^. These results suggest disparities in CR implementation in different regions of the country, although further investigation will be needed to clarify this aspect based on the waiting list of CR and participation in programs in rural versus urban areas. Furthermore, despite the fact that CR is covered by public health insurance in Japan for up to five months, the mean CR program length was roughly one month in both inpatient and outpatient settings. This length is much shorter than the six-month follow-up period set as an inclusion criterion for studies that provide high-quality evidence of the efficacy of CR^8,44^.

While most CR programs around the world include pharmacological and nutritional education as core components, their implementation in real-world settings has not been clarified^45^. Our study found that over 90% of CR participants were provided with pharmacological education, whereas only two-thirds were provided with nutritional education. Although the proportion of nutritional education provided to elderly individuals and women was lower than men, this result was reversed after adjusting for other covariates in the multivariable analysis. This is probably due to the fact that women are more likely to be elderly than men, and the proportion of critically ill patients with more comorbidities is higher among elderly individuals, i.e., they are more likely to be admitted to larger hospitals (which are more likely to accept such patients). Moreover, the patient to dietitian ratio is generally higher in larger hospitals, which could mean that it is more difficult to provide individualized services, such as nutritional guidance. This finding might be related to the scarcity of nutrition professionals working in a CR team. Further studies to clarify the association between care aspects and health care professionals are warranted.

The strengths of this study include the large sample size, generalizability of the results to the Japanese population, and detailed information on clinical practice due to the use of NDB data. However, there are also several limitations worth noting. First, we did not exclude patients who died in hospitals, as these patients could not be accurately identified in the database. This may have resulted in an overestimation of the number of patients who were considered eligible for CR. Second, there may have been other factors associated with CR participation which were not examined in this study, such as income status of patients and smoking history^46^. Third, detailed hospital- and community-level information could not be obtained due to limitations of the database. For example, factors such as auto-referral systems may affect CR participation^47^. Finally, since the present study used claims data, services not reimbursed by national health insurance, such as home-based CR and pharmacological and nutritional education by health care professionals other than pharmacists and dietitians, were not included in the present analysis, which might have led to underestimation of CR implementation and participation.

## Conclusion

In Japan, participation in CR after PCI or CABG is limited, with only one-third of eligible patients participating in CR and for an insufficient duration. Patients who underwent CABG were more likely to participate in CR than those who underwent PCI. Our findings suggest that there is room for improvement in the provision of nutritional education as part of CR, and that there is a need to promote CR implementation in the future.

## Supplementary Information

Supplementary Information.

## Data Availability

The dataset analyzed in this study is not publicly available and can only be accessed by people approved by the Ministry of Health, Labour and Welfare in a limited environment, but is available from the corresponding author upon reasonable request.
