# Supplementary material for: A nationwide survey on participation in cardiac rehabilitation among patients with coronary heart disease using health claims data in Japan
Source: Sci Rep. 2021 Oct 11;11:20096. doi: 10.1038/s41598-021-99516-1 (PMC8505519; doi:10.1038/s41598-021-99516-1)
Supplement: Supplementary file 1 — Supplementary Information. [file 41598_2021_99516_MOESM1_ESM.docx]

# Supplementary Table S1. Regional variation in proportions of hospitals providing cardiac rehabilitation

| Region | Hospitals providing PCI or CABG, n* | Hospitals providing cardiac rehabilitation, n | Proportion of hospitals providing cardiac rehabilitation, % |
| --- | --- | --- | --- |
| Hokkaido | 85 | 56 | 65.9 |
| Tohoku | 98 | 59 | 60.2 |
| Kanto | 388 | 285 | 73.5 |
| Chubu | 257 | 190 | 73.9 |
| Kinki | 271 | 196 | 72.3 |
| Chugoku | 78 | 63 | 80.8 |
| Shikoku | 54 | 37 | 68.5 |
| Kyushu | 192 | 161 | 83.9 |
| All | 1423 | 1047 | 73.6 |

*PCI: percutaneous coronary intervention, CABG: coronary artery bypass grafting
